# Pneumococcal Arthritis

**Published:** 1905-03-18

**Authors:** 


### PNEUMOCOCCAL ARTHRITIS.

Dr. L. Nattan-Larrier[1] has lately described a curious case of purulent arthritis occurring in a newly-born infant. The details are as follows:—The child was born at term, with a hare-lip, for which an operation was undertaken on the fourth day of life. Union was incomplete, and the wound suppurated. A week later the child showed constitutional signs of ill-health, had diarrhœa and sickness, and lost flesh. Sixteen days after the operation signs of pain in the right shoulder made their appearance, and were followed in the course of a day or two by swelling of the shoulder and fluctuation deeply seated about the joint. Nothing appears to have been done, and the infant died a day or two later. At the autopsy the joint was found to be full of the thick greenish pus characteristic of the pneumococcus, and bacteriological examination left no doubt as to the identity of the causal organism. We need not follow the author into an ingenious but unconvincing attempt to correlate the choice of the right shoulder for infection with a hypothetical injury during a somewhat protracted but otherwise normal labour, but the joint lesion is not infrequent, nor as the author asserts, a rarity in children, in this country at all events.

Dr. Nattan-Larrier has collected accounts of 35 cases, in 34 of which adults were affected, and remarks that it is in adults particularly that pneumococcal pyo-arthritis occurs in spite of the frequency of pneumococcal infections in childhood. But there is no doubt that the condition is quite common in childhood, and particularly in the early years of life. In the *Lancet*, August 1st, 1903, occurs an account of five cases in which the age of the patient was in four of the five cases below two years, and in two cases below one year. Other isolated instances have also been recorded, and there is no room for doubt that if bacteriological examination of the pus in cases of purulent arthritis in young children were more universally carried out, it would appear that the bulk of them depend upon the pneumococcus.

This variety of arthritis presents certain clinical features which in most cases are sufficient to make the diagnosis almost independent of bacteriological investigation. In the first place, the very youth of the victim is an argument in favour of a pneumococcal origin, for tuberculous joint disease (excepting only disease of the inter-phalangeal joints), is rarer in children under two than is the pneumococcal form. Again, while the pneumococcal arthritis gives rise to constitutional symptoms more marked than the ordinary tuberculous lesion, its constitutional evidences fall far short of the gravity commonly associated with a streptococcal infection, or of an infection with the staphylococcus pyogenes aureus. Yet another point of distinction is the appearance of the affected joint and the character of the fluctuation. In pneumococcal arthritis the joint affected—usually one of the biggest, *i.e.* hip, knee, shoulder, or elbow—is swollen and tender, and not uncommonly the skin over it is glazed and red, but there is an absence of cellulitis in the surrounding structures which gives the fluctuation obtained a sense of superficiality quite foreign to that attending an infection with the more usual pyogenic cocci. In this respect the character of the joint is more suggestive of a "cold" tuberculous abscess. The distinction between the tuberculous and the pneumococcal varieties depends to some extent, as has been said, upon the age of the patient, but at the same time the shortness of the history in the case of a pneumococcal lesion is generally sufficient to mark it out.

As regards the source of the infection, the French author ascribes a large majority to antecedent pneumococcal infections of the lungs. This is not so uniformly the case in childhood, for in some cases the joint lesion may be quite untraceable to any pulmonary or other lesion, while in not a few a primary pneumococcal otitis media appears to supply the organism to the joint.

The disease is undoubtedly grave: of Dr. Nattan-Larrier's cases 75 per cent. proved fatal, and of the other five cases referred to 80 per cent. died.

[1] Archives Gen. de Med., Feb. 28, 1905.

